# Editorial: Regulation of Vascular Function by Circulating Blood

**DOI:** 10.3389/fphys.2019.00492

**Published:** 2019-05-16

**Authors:** Joseph M. Rifkind, Dan E. Berkowitz, Joy G. Mohanty

**Affiliations:** ^1^Department of Anesthesiology, Johns Hopkins University, Baltimore, MD, United States; ^2^Department of Anesthesiology and Perioperative Medicine, University of Alabama Birmingham School of Medicine, Birmingham, AL, United States; ^3^Section of Molecular Dynamics, National Institute on Aging, Baltimore, MD, United States

**Keywords:** vasculature, endothelial cells, erythrocytes, blood flow, nitric oxide

The endothelium, which lines the inner surface of the vasculature, plays a crucial role in vascular function. Endothelial dysfunction results in hypertension, thrombosis, and inflammation that can lead to vascular diseases. Since the endothelium is constantly in contact with the blood and all its components including leukocytes, erythrocytes, and platelets, the flowing blood and the various components of the blood can influence endothelial function. In addition to effects involving individual components of the blood, the flowing blood affects the shear stress that the endothelium experiences, which induce cellular signaling that alters membrane potential and activates kinases. Blood flow, which is essential for the transport of oxygen, carbon dioxide, and other components to and from the tissues involves a cell free layer adjacent to the endothelium in addition to dilatation and constriction which is regulated by interactions with the endothelium. This research topic has investigated the different factors involving the blood, which affect vascular function.

In one study Tripathi et al. discusses autoregulation, which maintains constant blood flow. At low levels of blood pressure, the flow increases when the blood pressure increases. However, above a blood pressure of 70 mmHg autoregulation maintains constant blood flow in peripheral arteries. Three other studies discussed how changes taking place in the vasculature bypass autoregulation that maintains a constant blood flow. In one of these, Chatterjee investigated endothelial mechanotransduction whereby the shear stress and changes in flow patterns are sensed by the endothelium, which results in triggering biochemical signals that alter membrane potential and activate kinases. This process generates oxidant/redox signals that induce inflammation compromising proper vascular function. In a second study, Yalcin et al. discussed the endothelial glycocalyx layer that lines the endothelium. They demonstrated how degradation of the endothelial glycocalyx layer decreased the thickness of the cell free layer, and increased erythrocyte interactions with the vasculature affecting wall shear stress. A third study, Buerk et al. showed how the hypoxic conversion of tissue nitrite into nitric oxide can generate hypoxic vasodilation and improved blood flow. Several other studies (see below) have investigated the generation of NO by erythrocyte nitrite reduction. This study, however, emphasizes the role of the nitrite reduction reaction that occurs in tissues.

The erythrocyte is the dominant component of the blood and is responsible for the transport of oxygen. Several studies investigated how changes in the hemodynamic functionality of erythrocytes affect the interactions of the erythrocytes with the vasculature and how this affects the transport of oxygen through the circulatory system. In one study, Namgung et al. showed how deformability of erythrocytes affect the cell free layer that generally lines the vascular walls facilitating the flow of blood through the microcirculation. It was demonstrated that under physiological conditions RBCs with deformed cells preferentially flow near the vessel wall of small arterioles. In addition, the near wall accumulation of the less deformable cells near the walls of the vessel increases when passing by a bifurcation. In a second study, Barshtein et al. investigated transfused blood with impaired hemodynamic functionality. They found that with transfused blood there was a change in skin blood flow and a change in the hemoglobin level indicating that the transfused blood does affect blood flow in the microcirculation.

A number of other studies addressed interactions with the endothelium involving reactions taking place in erythrocytes. In one paper, Helms et al. discussed how the hypoxic vasodilatory effect is at least in part triggered by the release of NO, adenosine triphosphate and S-nitrosothiols from the erythrocyte under hypoxic conditions. They also discussed the effect of cell free hemoglobin generated by cell lysis that scavenges NO leading to vaso-constriction. In a second paper, Rifkind et al. investigated nitric oxide and reactive oxygen species generated by the erythrocyte. They demonstrated that these molecules can be released from the erythrocyte and transferred to the vasculature. Other studies have discussed the erythrocyte production of NO by nitrite reduction and the formation of reactive oxygen species by hemoglobin autoxidation. However, reacting with hemoglobin and antioxidant enzymes in the erythrocyte neutralizes the bulk of these substances. This paper, however, demonstrated that an unusually high affinity of partially oxygenated hemoglobin with band 3 of the erythrocyte membrane, together with a hypoxic enhanced nitrite reduction and hemoglobin autoxidation of this membrane associated partially oxygenated hemoglobin, triggers the release of these substances from the erythrocyte and, under proper conditions, their transfer to the vasculature. In the vasculature, the NO induces a vasodilatory effect and the reactive oxygen species can induce inflammation and other oxidative reactions that impair vasculature function. Namgung et al., as discussed above, showed how altered deformability of the erythrocyte impairs oxygen transport. They, however, did not discuss how erythrocytes with impaired deformability are produced. This is discussed in a third study by Diederich et al. on erythrocyte reactions. This study investigated hypertensive patients and found impaired deformability of erythrocytes and increased reactive oxygen species. They also found that tert-butylhydroperoxide decreased erythrocyte deformability. In addition, they reported that mice deficient in antioxidant/reducing enzymes were affected by tert-butylhydroperoxide to a greater extent than normal mice. They also found that NO did not affect deformability although it did minimize the effect of tert-butylhydroperoxide.

Two additional studies investigated the effects generated by the damage to the erythrocyte. In one paper, Said et al. discussed microparticles formed during erythrocyte maturation and in response to injury. These microparticles promote coagulation, endothelial adhesion, immune modulation and inhibit nitric oxide bioavailability. These changes significantly disturb effective oxygen delivery. In a second paper, Jana et al. investigated the effect of cell free hemoglobin generated during hemolytic episodes in the circulation. The effects were most dramatic for hemoglobins, HbS, and HbE associated with sickle cell disease and beta–thalasemia respectively. These hemoglobins readily undergo oxidation to metHbs as well as ferryl hemoglobin. These proteins damaged the integrity of the endothelial monolayer with a rise in reactive oxygen species, lipid hydroperoxide and increased expression of oxidative stress proteins. In addition, a rise in uncoupled mitochondrial respiration took place.

In reviewing how vascular function is affected by circulating blood, many aspects involving the interactions between the blood and the vasculature were investigated. We started with studies involving autoregulation and how it is generated and maintained. The role of hypoxia in maintaining blood flow by increased generation of NO was also investigated. Since the erythrocyte is responsible for the transport of oxygen, a major part of our review involved the erythrocyte. Several studies investigated hemodynamic functionality under normal conditions and how this can be impaired. In dealing with the erythrocyte, it was also necessary to discuss reactions taking place within the erythrocyte that influence the vasculature as well as effects associated with particles or molecules released from the erythrocyte. These studies provide an important insight into being able to understand the various factors that regulate blood flow and the transport of oxygen and other substances to and from the tissues.

While this review deals with the important role of nitric oxide, the cell free layer and mechanotransduction and how these factors are affected by the erythrocyte, the crucial roles of leukocytes and platelets in affecting vascular function was not addressed. In any future study these need to be addressed.

## Author Contributions

JR has written the complete editorial. DB reviewed and approved the manuscript. JM contributed to editing and corrections.

### Conflict of Interest Statement

The authors declare that the research was conducted in the absence of any commercial or financial relationships that could be construed as a potential conflict of interest.

